# The Effect of Oral Vaccination with *Mycobacterium bovis* BCG on the Development of Tuberculosis in Captive European Badgers (*Meles meles*)

**DOI:** 10.3389/fcimb.2017.00006

**Published:** 2017-01-24

**Authors:** Mark A. Chambers, Frank Aldwell, Gareth A. Williams, Si Palmer, Sonya Gowtage, Roland Ashford, Deanna J. Dalley, Dipesh Davé, Ute Weyer, Francisco J. Salguero, Alejandro Nunez, Allan K. Nadian, Timothy Crawshaw, Leigh A. L. Corner, Sandrine Lesellier

**Affiliations:** ^1^Department of Bacteriology, Animal and Plant Health AgencyAddlestone, UK; ^2^Immune Solutions Ltd, University of OtagoDunedin, New Zealand; ^3^Animal Services Unit, Animal and Plant Health AgencyAddlestone, UK; ^4^Department of Pathology, Animal and Plant Health AgencyAddlestone, UK; ^5^School of Veterinary Medicine, Faculty of Health and Medical Sciences, University of SurreyGuildford, UK; ^6^Animal and Plant Health AgencyStarcross, UK; ^7^School of Veterinary Medicine, University College DublinDublin, Ireland

**Keywords:** badgers, *Mycobacterium bovis*, oral BCG, tuberculosis, vaccine

## Abstract

The European badger (*Meles meles*) is a reservoir host of *Mycobacterium bovis* and responsible for a proportion of the tuberculosis (TB) cases seen in cattle in the United Kingdom and Republic of Ireland. An injectable preparation of the *bacillus Calmette-Guérin* (BCG) vaccine is licensed for use in badgers in the UK and its use forms part of the bovine TB eradication plans of England and Wales. However, there are practical limitations to the widespread application of an injectable vaccine for badgers and a research priority is the development of an oral vaccine deliverable to badgers in bait. Previous studies reported the successful vaccination of badgers with oral preparations of 10^8^ colony forming units (CFU) of both Pasteur and Danish strains of BCG contained within a lipid matrix composed of triglycerides of fatty acids. Protection against TB in these studies was expressed as a reduction in the number and apparent progression of visible lesions, and reductions in the bacterial load and dissemination of infection. To reduce the cost of an oral vaccine and reduce the potential for environmental contamination with BCG, it is necessary to define the minimal efficacious dose of oral BCG for badgers. The objectives of the two studies reported here were to compare the efficacy of BCG Danish strain in a lipid matrix with unformulated BCG given orally, and to evaluate the efficacy of BCG Danish in a lipid matrix at a 10-fold lower dose than previously evaluated in badgers. In the first study, both BCG unformulated and in a lipid matrix reduced the number and apparent progression of visible lesions and the dissemination of infection from the lung. In the second study, vaccination with BCG in the lipid matrix at a 10-fold lower dose produced a similar outcome, but with greater intra-group variability than seen with the higher dose in the first study. Further research is needed before we are able to recommend a final dose of BCG for oral vaccination of badgers against TB or to know whether oral vaccination of wild badgers with BCG will significantly reduce transmission of the disease.

## Introduction

Tuberculosis (TB) in cattle, arising from infection with the bacterium *Mycobacterium bovis*, causes substantial financial losses to the governments of the United Kingdom, Ireland and the cattle industry (University of Reading, 2004). In these countries, the European badger (*Meles meles*) is a wildlife reservoir of *M. bovis* that can spill over into cattle, frustrating attempts to control the disease using measures focussed on cattle alone (Brooks-Pollock et al., 2014). Controlling infection within badger populations at risk of transmitting the disease to cattle is difficult, expensive, and contentious, especially control by culling. Reducing the prevalence of TB in badgers through vaccination, and as a consequence reducing the risk of transmission of infection to and from cattle, is part of the long-term plan to eradicate TB from cattle in the UK and Ireland.

The BCG (*bacillus Calmette-Guérin*) vaccine, an attenuated strain of *M. bovis*, is currently the only vaccine available for use in badgers. It is licensed in the UK as a lyophilized preparation for the intramuscular vaccination of badgers (BadgerBCG, 2010). The costs associated with its purchase and the costs and practical limitations associated with its administration limit its use in the field (Enticott et al., 2012). The best prospect for the wide-scale use of BCG in badgers relies on the development of an oral presentation of BCG in a bait matrix for ingestion by badgers.

In previous studies conducted by University College Dublin, we reported the successful vaccination of badgers with an oral preparation of BCG contained within a protective lipid matrix composed of triglycerides of fatty acids (Corner et al., 2010; Murphy et al., 2014). This matrix provided a stable oral bait for BCG vaccine (reviewed in Beltrán-Beck et al., 2014). The Pasteur strain of BCG at an oral dose of 10^8^ colony-forming units (CFU) was used in the first of these studies: the Pasteur strain was recommended by a joint WHO/FAO/OIE consultative group in 1994 for vaccine trials in animals (World Health Organization, 1994). Protection against TB was expressed as fewer organs/tissues with visible lesions, a decrease in the severity of visible lesions, fewer sites of infection, and lower bacterial load in the lungs and thoracic lymph nodes in vaccinates compared with controls. Danish 1331 strain is the only BCG vaccine currently licensed in the European Union for use in humans for immunization against TB and is available commercially from the Staten Serum Institute, Denmark. It is also the strain of BCG used in BadgerBCG. Basing an oral BCG badger vaccine on the Danish 1331 strain therefore would simplify licensing. When the efficacy of these two BCG strains in lipid matrix at 10^8^ CFU was tested in badgers, they gave similar levels of protection (Murphy et al., 2014).

Previous studies in mice, guinea pigs and possums showed oral encapsulated BCG gave superior protection to unformulated oral BCG (Clark et al., 2008). We wanted to make this comparison in badgers. Also, in previous oral vaccine studies in badger we used BCG at more than 10 times the injectable dose and some excretion of BCG in feces was observed (Corner et al., 2010). From the perspective of reducing both the cost of an oral vaccine and the potential for environmental contamination with BCG, we need to define the minimal efficacious dose for badgers. Given that the effective dose of injected BCG appears to bear no relationship to the size of the target animal—10 equivalent human doses of BCG Danish for badgers (Lesellier et al., 2011) compared to 0.5 equivalent human doses for cattle (Buddle et al., 2013)—these data have to be generated empirically using badgers. The objectives of the two vaccine efficacy studies (VES) reported here were to compare the efficacy of BCG Danish strain in a lipid matrix with unformulated BCG given orally (VES3) at a dose of 10^8^ CFU, and to evaluate the efficacy of BCG Danish in a lipid matrix at a 10-fold lower dose than previously evaluated in badgers (VES4).

We report that the incorporation of BCG in the lipid matrix appears beneficial for effective oral delivery, and encouraging results were obtained with unformulated oral BCG. In a second study, promising results were obtained with 10^7^ CFU BCG in the lipid matrix but, in contrast to the first study, not with 10^8^ CFU BCG in the lipid matrix. These studies have important implications for the progress of work toward a reproducibly efficacious oral vaccine against TB in badgers.

## Materials and methods

Two separate studies were conducted to evaluate the protective effect of different BCG oral vaccines in badgers subjected to endobronchial challenge with virulent *M. bovis*; an established model for what is primarily a respiratory disease in wild-infected badgers. During the studies, immune responses were measured. Clinical samples, as detailed below, were collected to look for excretion of BCG and *M. bovis*. The burden of disease in the badgers was ascertained at the end of each study on the basis of pathology (visible lesions, histopathology) and bacteriology.

### Ethics statement

All animal procedures were covered by licenses issued by the UK Home Office under the Animal [Scientific Procedures] Act 1986, and approved by the Animal Welfare and Ethical Review Board at the Animal and Plant Health Agency (APHA). This manuscript was prepared to comply with the ARRIVE Guidelines for reporting animal research (Kilkenny et al., 2010).

### Badgers

Thirty-seven wild badgers of both sexes and mixed age groups were obtained from counties in England where the incidence of TB in cattle and badgers was historically either very low or absent. Three additional badgers were born in captivity at APHA. The wild badgers were allowed to acclimatize to captivity for a week. All animals used in experiments were confirmed as free of TB on the basis of three negative IFNγ tests and negative for *M. bovis* on culture of clinical samples (tracheal aspirate, laryngeal swab, urine, rectal swab and feces) taken on three separate occasions approximately 30 days apart before enrolment into a study. Badgers were housed in isolation from wild badgers in the Natural Environment Centre (NEC) in groups of four to a pen, as far as possible corresponding to their original social grouping and then transferred to a Containment Level 3 (CL3, Advisory Committee on Dangerous Pathogens) animal facility in the same social groups. Allocation of pen groups to treatments was by randomisation. Twenty animals were enrolled into each study. In VES3, the badgers were transferred from the NEC to the ACDP CL3 facility before vaccination. In VES4, they were moved to the CL3 facility 70 days after vaccination (20 days in advance of the challenge). Vaccination was conducted in summer (June-July) and the challenge in autumn (September-October) and the studies were terminated in December-January.

Each badger was given a unique identifier (microchip AVID PLC, Uckfield, East Sussex) and by tattooing the last three digits of the microchip number on the abdomen. Throughout the studies, the badgers received dog food and peanuts daily, occasionally chicken eggs from the APHA Specific-Pathogen-Free hatchery, and they were free to forage in the NEC. The badgers were examined visually on a daily basis inside wooden sett boxes provided to them. Tap water was provided *ad libitum*.

### CB-BCG vaccine

A suspension of BCG Danish strain 1131 was supplied by the Statens Serum Institute, Denmark in a solution of 1.5% (w/v) sodium 2-aminopentanedioate (monosodium glutamate). The vaccine [Concentrated Bulk (CB)-BCG] was stored in 100 μl aliquots at −10.5 to −29.5°C. At the time of use, the vaccine had been in storage for approximately 25 weeks. The titre of CB-BCG (Table 1) was determined from the thawed stock of vaccine on the day of administration.

**Table 1:** **Vaccination of badgers with BCG: number of animals in each experiment, treatment group, sex, and the vaccine and challenge doses**.

**Experiment**	**Treatment group**	**Dose of BCG (CFU)**	**Dose of *M. bovis* (CFU/ml)**	**Number of badgers**	**Sex distribution (M, F)**
VES3	CB-BCG	9.30 × 10^7^	1.20–1.85 × 10^3^	4	3M, 1F
	HD BCG-Liporale™	1.86 × 10^8^		8	3M, 5F
	Control	–		8	4M, 4F
VES4	HD BCG-Liporale™	3.20 × 10^8^	0.98–1.01 × 10^3^	4	1M, 3F
	LD BCG-Liporale™	9.65 × 10^6^		7	2M, 5F
	Control	–		8	4M, 4F

### BCG-liporale™ vaccine

*Bacillus Calmette-Guérin* (BCG) Danish strain 1131 was supplied by Immune Solutions Ltd (ISL), University of Otago, New Zealand in a lipid matrix. Unlike CB-BCG, the vaccine (BCG-lipid PK/Liporale™) was prepared from a fresh, broth-grown culture of BCG and stored according to standard protocol (Aldwell et al., 2003). Stocks were frozen at −80°C; a temperature at which the viability of similarly prepared BCG stocks had been found to be stable for 24 months (Frank Aldwell, personal communication). Vaccine was prepared from frozen batches of BCG, as follows. The BCG pellets were thawed for 30 min at 20–22°C, re-suspended and dispersed in lipid PK (C1758; Sigma-Aldrich, Australia Ltd) that had been warmed to 26°C in a stirred water bath. Lipid formulated BCG was gently mixed in 50 ml tubes (Falcon) and allowed to cool to 22°C. 1.0–1.2 ml volumes were taken up into 3 ml Luer-Lock syringes (Becton Dickinson) and capped with either blue or white Combi-lock plugs (Codan) according to the concentration of BCG in the preparation: 2–3 × 10^8^ CFU/ml (HD, VES3, and VES4) or 10^7^ CFU/ml (LD, VES4) (Table 1) per ml. Capped syringes were transferred to 4°C. These were consigned to APHA as perishable goods (cold chain) and maintained at <8°C, as determined by a temperature monitor located with the samples. On receipt at APHA, the syringes were maintained at 4°C until use. Additional syringes of Liporale™ matrix alone were supplied as negative controls. Representative syringes containing vaccine were retained by ISL for retrospective analysis of CFU. As Liporale™ is intended to be both vaccine vehicle and bait matrix, a volume of 1 ml of BCG-Liporale™ or Liporale™ (placebo) was administered.

### Mycobacterium bovis

The *M. bovis* strain used for challenge was originally isolated from a wild badger in the UK in 1997 (isolate 74/0449/97). It was stored as a first passage stock culture until expanded and then stored as frozen aliquots (−80°C). The clonality of the culture was confirmed by demonstrating the spoligotype–SB0140 (APHA type 9) and VNTR type (8-5-5-5-3-3.1) of 27 individual colonies (10% of those grown from a 1:1000 dilution of a culture at approximately 10^5^ CFU/ml). The stock vials used for the challenge had not been passaged further. Each vial contained approximately 10^7^ CFU/ml viable *M. bovis*. On the day of challenge, one aliquot was thawed, serially diluted in sterile water + 0.05% (v/v) Tween 80 to contain approximately 10^4^ CFU/ml. The last dilution to 1000 CFU/ml was made in PBS + 0.05% (v/v) Tween 80. This solution was vortexed immediately before delivery to diminish the risk of bacterial clumping. The titre of the challenge inoculum was determined by plating on modified Middlebrook 7H11 agar (Gallagher and Horwill, 1977) a sample from a syringe kept in the same conditions as those used for challenge.

### Clinical sampling

Badgers were anesthetized once every 2 to 3 weeks by intramuscular injection of approximately 10 mg/kg of ketamine (Vetalar®, Pfizer Animal Health, New York, NY, USA), 100 μg/kg of medetomidine (Domitor®, Pfizer Animal Health) (Davison et al., 2007) and 100 μg/kg of butorphanol (Torbugesic®, Zoetis UK Ltd, Tadworth, Surrey, UK). Blood was collected by jugular venipuncture into heparinised and serum separation BD Vacutainer® tubes for immunological assays. Body weight, general condition and rectal temperature were recorded for welfare monitoring purposes. Tracheal mucus was collected by aspirating with a flexible urinary catheter and dispensed into Middlebrook 7H9 broth supplemented with ADC enrichment (BD, Oxford, UK). Laryngeal and rectal swabs were collected and placed into 7H9 broth and PBS, respectively. Urine was collected into sterile 15 ml plastic tubes following manual compression of the bladder.

### Vaccination and challenge

*Bacillus Calmette-Guérin* (BCG) Liporale™ and the Liporale™ placebo were administered to anesthetized badgers using a syringe and catheter to the proximal region of the esophagus. In VES3, BCG-Liporale™ and Liporale™ placebo were heated to 37°C degrees before delivery and were fully liquefied. In VES4, the BCG-Liporale™ and Liporale™ placebo were heated to 24°C degrees before delivery and was an opalescent semi-solid. After vaccine or placebo administration the syringe was removed from the catheter and 10 ml of air was flushed through the catheter to ensure that all vaccine and placebo was delivered. After treatment, the badgers were kept in sternal recumbence until they had recovered from anesthesia. ISL determined the concentration of BCG in material withheld but treated in the same way as that delivered to the APHA (Table 1).

CB-BCG was thawed and gently mixed immediately prior to use and was administered to each badger as a 180 μl volume deposited on the surfaces of the left and right tonsils. If CB-BCG was to be used for widespread application it would most likely be inside oral bait, so this smaller volume was chosen as representative of the volume that might be used, and to achieve a dose of 1 × 10^8^ CFU, directly comparable to the ISL lipid.

*Mycobacterium bovis* challenge was performed under anesthesia 13 weeks after vaccination by endobronchial instillation of 1 ml of *M. bovis* suspension per badger using a fiberscope (Olympus URF P2 3.6 mm outside diameter, 1.8 mm inside diameter, 70 cm long), targeting the bronchus of the right middle lobe. For each animal the *M. bovis* suspension was inoculated via a sterile plastic catheter (1.5 mm diameter) and the catheter was flushed with 1 ml PBS. Between animals, the fiberscope was disinfected with ortho-phthalaldehyde and 70% (v/v) ethanol then rinsed with sterile water. The used catheters were discarded.

Table 1 shows the number and sex of animals in each treatment group and study, and the doses of BCG vaccine and *M. bovis* administered.

### Bacteriology

Clinical samples (tracheal aspirate, laryngeal swab, urine and rectal swab) were taken every 2–3 weeks after vaccination and challenge to detect BCG or *M. bovis* excretion. The presence of BCG excreted per pen/treatment group was also measured in feces collected in the pens for 2 weeks after vaccination.

All samples were cultured on the day of collection, except for rectal swabs and feces that were stored at 2–8°C in saline solution and then cultured on the following working day. The laryngeal swab and tracheal aspirate were agitated in 7H9 broth and then cultured on six modified Middlebrook 7H11 slopes containing OADC enrichment (BD) and incubated for 12 weeks at 37°C. After challenge, when samples could contain both BCG and *M. bovis, two* additional plates of solid Middlebrook 7H11 medium containing OADC and 60 μg/ml cycloserine; the latter to select for BCG growth (Rist et al., 1967; Grange et al., 1996), were also used per sample. Except for fecal swabs and feces, the samples were not decontaminated before culture. Faecal swabs and feces samples were dispersed in 0.85% (w/v) saline solution. The following working day the swab was discarded and the saline solution decontaminated with 5% (v/v) final volume ethanedioic acid (oxalic acid) for 20–30 min at room temperature. The oxalic acid was removed by a wash step using saline and material for sowing was retrieved by centrifugation.

Tissue samples collected at post-mortem were collected aseptically, weighed and frozen at −20°C. The following tissues were sampled: left and right lung lobes (cranial, caudal, middle, accessory); LNs, left and right where appropriate (anterior and posterior mediastinals, left and right bronchial, mandibular, parotid, retropharyngeal, axillary, inguinal, popliteal, hepatic, mesenteric); tonsils; mediastinal pleura; spleen; liver; heart; and kidneys. Tissues for culture were thawed at room temperature and each tissue sample was cultured separately except for pooled left and right axillary LN, pooled left and right inguinal LN and pooled left and right popliteal LN. Left and right kidneys were also cultured together. Tissues were homogenized in 10 ml 0.85% (w/v) saline using IKA® DT-Tubes (IKA® Werke GmbH & Co. KG, Staufen, Germany) and a 100 μl of homogenate spread onto each culture plate. If contamination occurred, stored homogenate was re-cultured. Up to 20 colonies from one plate per tissue were typed by spoligotyping (Kamerbeek et al., 1997) and VNTR (Frothingham and Meeker-O'connell, 1998) to confirm that the isolates were the same as the challenge strain.

### Immunological assays

The immune responses were monitored by measuring the frequency of peripheral blood mononuclear cells (PBMC) producing IFNγ by ELISPOT assay (Lesellier et al., 2006), and by measuring the presence of specific antibodies in serum against a mixture of *M. tuberculosis* Complex antigens, including MPB83, using the Brock TB Stat-Pak test (Chambers et al., 2008). Antigens used to stimulate PBMC cultures were PPD-B (Weybridge antigen) and a cocktail of ESAT-6 and CFP-10 (kind gift of M. Singh, Lionex GmbH, Braunschweig, Germany).

### Post-mortem examination

All badgers were killed humanely with an intravenous overdose of sodium pentobarbitone 12 weeks after challenge with *M. bovis* and promptly subjected to post-mortem examination. Experience of this challenge model has shown that TB lesions have developed by this point but clinical signs of TB have not. During collection of tissues and organs their surface was examined for visible lesions. After collection lymph nodes (LNs) and organs (except lung) were sliced for examination of their internal structures. The surfaces of the lung were examined at collection, before samples were taken from the lung lobes for culture. At this point, the rest of the lung was immersed in formalin using a 1/10 (v/v) tissue/fixative ratio. The trachea was opened to ensure the lungs were in contact with formalin during the fixation. Paper towels were placed on top of the organ to avoid unfixed tissue due to floating. After 1 week of fixation in buffered formalin, the lungs were finely sliced. Samples of fresh tissues were collected for histopathology and culture. For large organs, such as spleen, approximately 3 × 3 × 3 cm of tissue was submitted for culture and a similar volume for histology. A visible lesion score was derived using a standardized ordinal scoring system from 0 no lesions, 1 (few foci or slight enlargement of the LN) through to 4 (extensive caseation or areas of coalesced foci) (Corner et al., 2007; Crawshaw et al., 2008). Only visible lesions in organs and LNs from which *M. bovis* was isolated by culture or were found to contain acid-fast bacilli (AFB) in Ziehl-Neelsen (ZN) stained histological sections counted toward the final score. The final visible lesion score was derived from the sum of the highest scoring lung lobe plus the scores from all other organs and LNs.

### Histopathology

Granulomas were scored for their severity or degree of maturation from 1 to 4. Those given a score of 1 contained lymphocytes, epithelioid and plasma cells. Those given a score of 4 were considered the most severe lesions with caseation and mineralization present. Tissue sections with active lymphoid follicles only, commonly seen in lymph nodes and spleen, were scored as 0 and therefore were not included in the calculation of final scores. More developed granulomas may be characterized by greater deposition of collagen within or around the granuloma (Wangoo et al., 2005). Collagen was visualized by Martius Scarlet Blue (MSB) staining, and its abundance scored from 0 to 2. The score was allocated from the most severe lesion observed on the section. Final scores for each animal were calculated as a sum of the individual tissue scores for granuloma and collagen. Scores were not included for a tissue from which *M. bovis* was neither cultured nor AFB present histologically.

### Data analysis

Raw data were entered into a Microsoft Access database by operatives blinded to the treatment each badger had received. The treatment allocations were revealed only when all data had been entered and scores calculated. For each study, analysis of disease severity scores, the number of tissues with visible lesions and histopathology lesions were conducted using the non-parametric Kruskal Wallis test. Significant differences between treatment-groups within each study were investigated further using Dunn's test for multiple pair wise comparisons, vaccination against control. Culture data were continuous and normally distributed and analyzed by parametric one-way ANOVA and investigated further using Sidak's multiple comparison post-test. Statistically significant differences (*p* < 0.05) are denoted in the figures as a bar. All other statistical analyses are described in context.

## Results

One VES4 badger vaccinated with LD BCG-Liporale™ died under anesthesia 4 weeks after challenge and was excluded from the assessment of that study. This badger did not present any pathological clinical sign and its weight and rectal temperature (12 kg and 39.6°C, respectively) were within the 10–90% percentile of the group.

### Tuberculous lesions post-mortem

**Figure 1:**
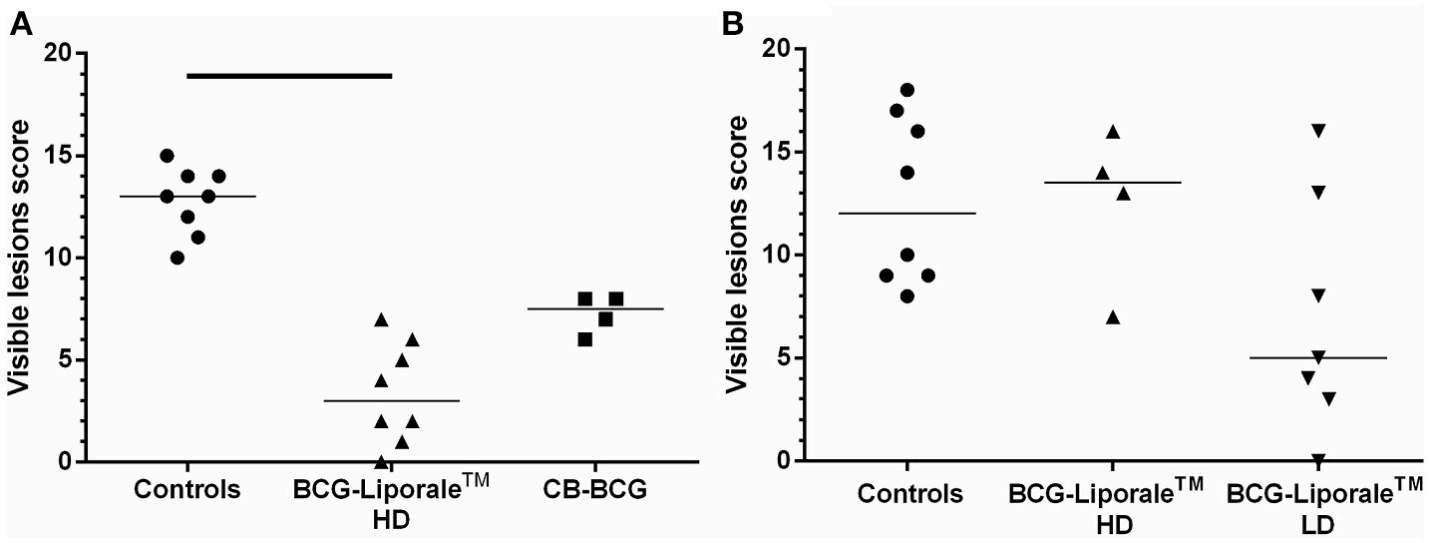
**Vaccination of badgers with BCG and challenged 13 weeks later with endobronchial ***M. bovis*****. Lesion scores at post-mortem (12 weeks post-challenge) in VES3 **(A)** and VES4 (**B**). Individual animal results are shown together with the group median. Badgers were vaccinated with either CB-BCG (■) or HD BCG-Liporale™ vaccine [denoted by the (▲)] or LD BCG-Liporale™ vaccine [denoted by the delta symbol (▼)]. Liporale™ alone was used as a negative control for vaccination (•). A significant difference according to Dunn's test for multiple pair wise comparisons is shown by the bar.

Only two badgers had no visible lesions, one vaccinated with HD BCG-Liporale™ in VES3 (Figure 1A) and the other vaccinated with LD BCG-Liporale™ in VES4 (Figure 1B). In most animals with visible lesions, the right lung and draining LNs (either the right tracheobronchial or the posterior mediastinal) contained the most severe lesions, which is consistent with the route of experimental infection. The most frequently affected LN outside of the thoracic cavity was the hepatic LN. No lesions were found in the kidneys of any animal, although *M. bovis* was isolated from the kidneys of one control animal in VES4 and from a CB-BCG vaccinated animal in VES3. In neither case was *M. bovis* isolated from the urine.

The sum of the visible lesion scores, a measure of disease severity, for each animal is shown in Figure 1. Control animals had a median score of 13 (range, 10–15) in VES3 (Figure 1A) and 12 (range, 8–18) in VES4 (Figure 1B), demonstrating the consistency of the challenge model despite these being outbred animals. Analysis of the accumulated visible lesion scores revealed a significant reduction in the median score for badgers vaccinated with HD BCG-Liporale™ (3) compared with controls in VES3 (13). The CB-BCG vaccine reduced the median lesion score to 7.5 and whilst this suggested a protective effect, the difference was not significant.

In VES4, the median lesion severity score for LD BCG-Liporale™ (5) was less than the controls (12) but the difference was not statistically significant. The median for HD BCG-Liporale™ of 13.5 was higher than the controls but the difference was not significant.

### Histopathology

**Figure 2:**
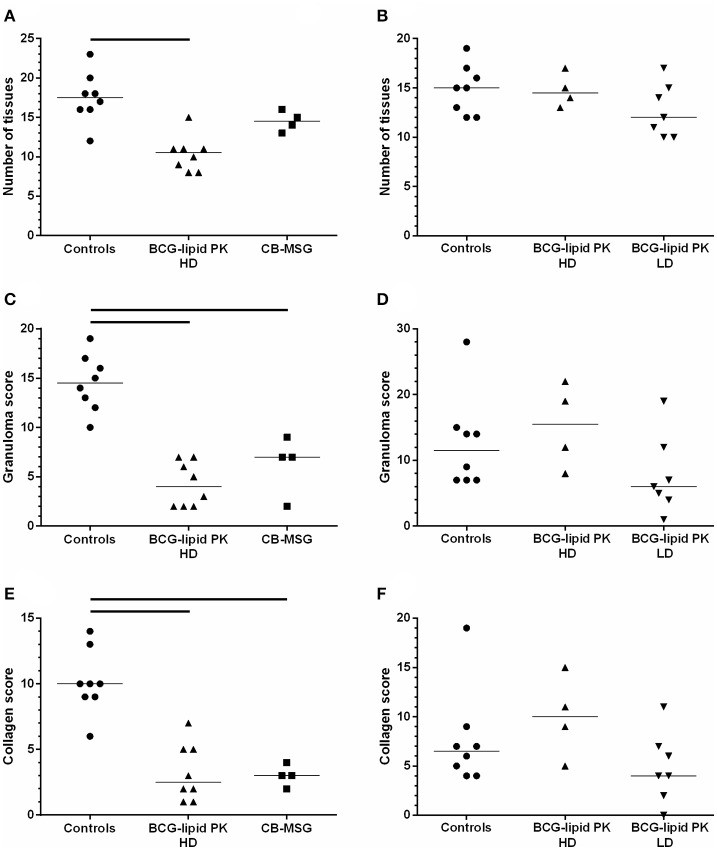
**Vaccination of badgers with BCG and challenged 13 weeks later with endobronchial ***M. bovis*****. Histopathology assessment. Number of sites with histological lesions **(A,B)**, scores for granuloma severity **(C,D)** and collagen **(E,F)** in VES3 (left panel) and VES4 (right panel). Individual animal results are shown together with the group median. Badgers were vaccinated with either CB-BCG (■), HD BCG-Liporale™ (▲) or LD BCG-Liporale™ vaccine (▼). Liporale™ alone was used as a negative control for vaccination (•). A significant difference according to Dunn's test for multiple pair wise comparisons is shown by the bar.

Three measures of disease severity using histopathological characteristics are shown for each animal in Figure 2. Consistent with the results for visible lesion scores, HD BCG-Liporale™ vaccination in VES3 significantly reduced the number of sites with histological lesions (Figure 2A), the severity of granulomas (Figure 2C), as well as degree of collagen deposition (Figure 2E). Vaccination with CB-BCG also significantly reduced the severity of granulomas (Figure 2C) as well as the degree of collagen deposition (Figure 2E). In VES4, in all three histological measures of severity (Figures 2B,D,F) the LD BCG-Liporale™ group had lower median scores than the control but these differences were not significant. In VES4, the HD BCG-Liporale™ vaccinates had higher median granuloma and collagen scores than the controls but the differences were not significant.

### Distribution of *M. bovis* infection

**Figure 3:**
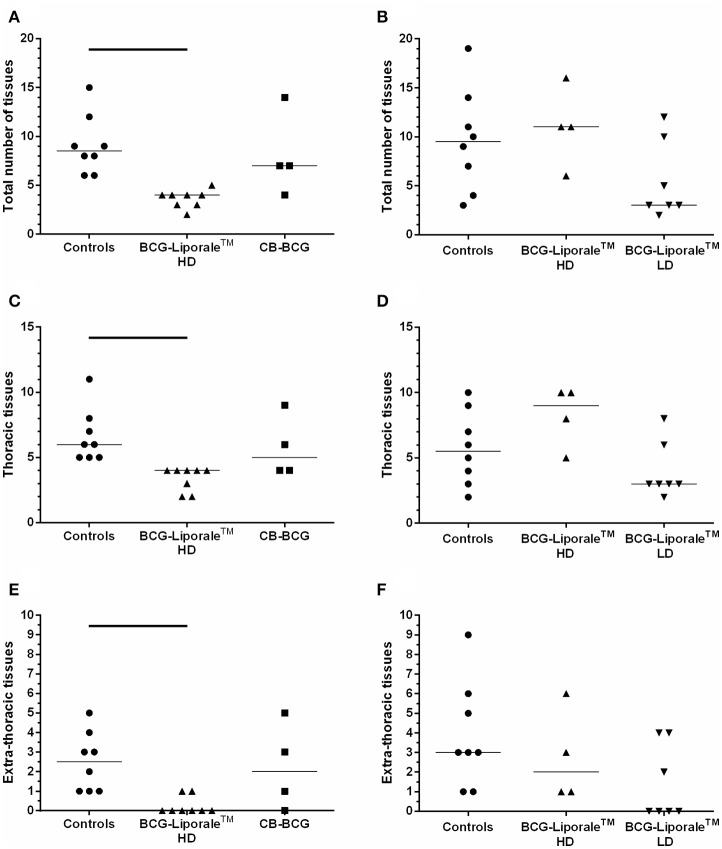
**Vaccination of badgers with BCG and challenged 13 weeks later with endobronchial ***M. bovis*****. Number of organs/tissues from which *M. bovis* was isolated or AFB found 12 weeks post-challenge for VES3 (left panel) and VES4 (right panel). The total number of affected tissues is shown **(A,B)**, together with their distribution: thoracic **(C,D)** or extra-thoracic **(E,F)**. Individual animal results are shown together with the group median. Badgers were vaccinated with either CB-BCG (■), HD BCG-Liporale™ (▲) or LD BCG-Liporale™ vaccine (▼). Liporale™ alone was used as a negative control for vaccination (•). A significant difference according to Dunn's test for multiple pair wise comparisons is shown by the bar.

The *M. bovis* challenge strain was the only wild-type strain recovered from badgers in these studies. The total number of sites affected by *M. bovis* on the basis of confirmed visible lesions is shown for each animal in Figure 3, along with sites of thoracic and extra-thoracic infection. In the control groups the median numbers of infected sites were 8.5 and 9.5 for VES3 and VES4, respectively (Figures 3A,B), i.e., approximately a third of all the organs/tissues examined. Only vaccination with HD BCG-Liporale™ in VES3 reduced this significantly to a median of four sites.

**Figure 4:**
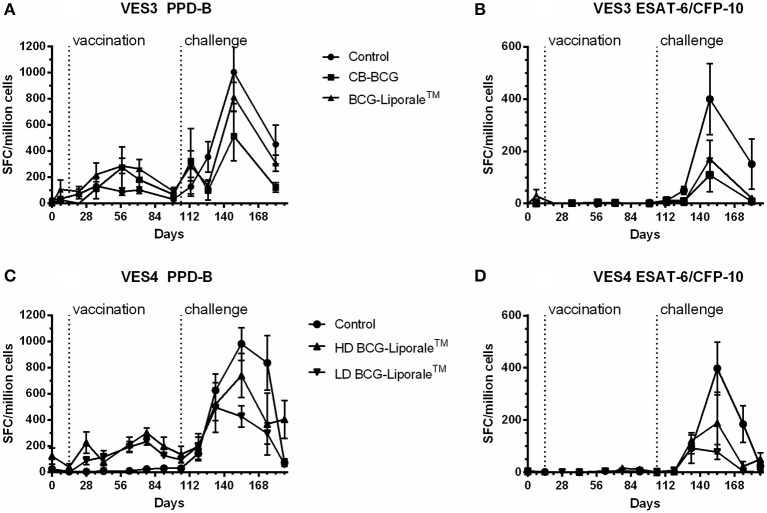
**Vaccination of badgers with BCG and challenged 13 weeks later with endobronchial ***M. bovis***: IFNγ ELISPOT results from experiment VES3 (A,B)** and VES4 **(C,D)** expressed as the net spot forming cells (SFC) per million cells in response to stimulation with PPD-B **(A,C)** or ESAT-6/CFP-10 **(B,D)**.

Vaccination altered the distribution of infection. In VES3, the number of sites infected in total, and separately in the thoracic cavity and extra-thoracic sites, were fewer after vaccination with both HD BCG-Liporale™ and CB-BCG than in the controls. However, the differences were only significant in the comparison between HD BCG-Liporale™ and the controls. In VES4, the number of sites infected in total, and separately in the thoracic cavity and extra-thoracic sites, were fewer after vaccination with LD BCG-Liporale™ than in the controls, although none of these differences were significant. In all control animals, *M. bovis* had disseminated from the lung, where it had been deposited, to extra-thoracic sites; with a range of 1–9 sites affected (Figures 3E,F). In contrast, in both studies vaccination with BCG-Liporale™, both LD and HD, reduced the dissemination of *M. bovis* from the lung to other organs, such that we were unable to detect *M. bovis* in any of the extra-thoracic sites in 6/8 animals vaccinated with HD BCG-Liporale™ in VES3 (Figure 3E) and 4/7 animals vaccinated with LD BCG-Liporale™ in VES4 (Figure 3F). These proportions were significantly lower than the controls in both studies (Fisher's Exact test).

### Bacteriology

*Mycobacterium bovis* was cultured from organs and tissues with macroscopic lesions and from tissues with only microscopic lesions, and also from organs and tissues without lesions (data not shown). *M. bovis* was isolated intermittently from clinical samples taken from animals after challenge. There were no significant differences between treatment groups in these respects (data not shown). No urine sample was positive.

*Bacillus Calmette-Guérin* (BCG) was only isolated from clinical samples twice and only in VES3 and from the same two badgers, both vaccinated with BCG-Liporale™. The isolates were from a laryngeal swab 2 weeks after vaccination and from a tracheal aspirate 4 weeks after vaccination. BCG was not isolated from feces collected from the animal pens. Post-mortem, BCG was isolated from the left retropharyngeal LN of one badger and from the right retropharyngeal LN of another, vaccinated 25 weeks previously with CB-BCG and BCG-Liporale™, respectively, in VES3.

### Immunology

In VES3 and VES4, an IFNγ response to PPD-B was detected by ELISPOT in all badgers after vaccination and in all badgers after challenge (Figures 4A,C). In VES3, responses to PPD-B were also seen in control animals prior to challenge (Figure 4A). In VES4, background responses of control animals to PPD-B were very low and there was a significantly greater response to PPD-B in those badgers receiving HD BCG-Liporale™ compared to LD BCG-Liporale™ 4 weeks after vaccination (Figure 4C), but at no other time point.

Vaccinated animals before challenge did not respond to a combination of ESAT-6 and CFP-10 antigens, consistent with the absence of genes encoding these antigens from BCG (Figures 4B,D). There was one exception, a single animal within the Liporale™ group that had a marginally elevated response to ESAT-6 and CFP-10 on day 7 before vaccination. However, the mean result for this group was statistically indistinguishable from the other treatment groups and an elevated response was not seen again at any other time point prior to challenge in this animal. In VES3, the peak mean response to ESAT-6/CFP-10 was seen 5 weeks after challenge in both the control and two vaccinated groups. In VES4, the peak mean response to ESAT-6/CFP-10 was seen 7 weeks after challenge in the control and HD BCG-Liporale™ groups but at 4 weeks in the LD BCG-Liporale™ group. In all cases, the level of IFNγ production correlated poorly with disease severity (data not shown).

**Figure 5:**
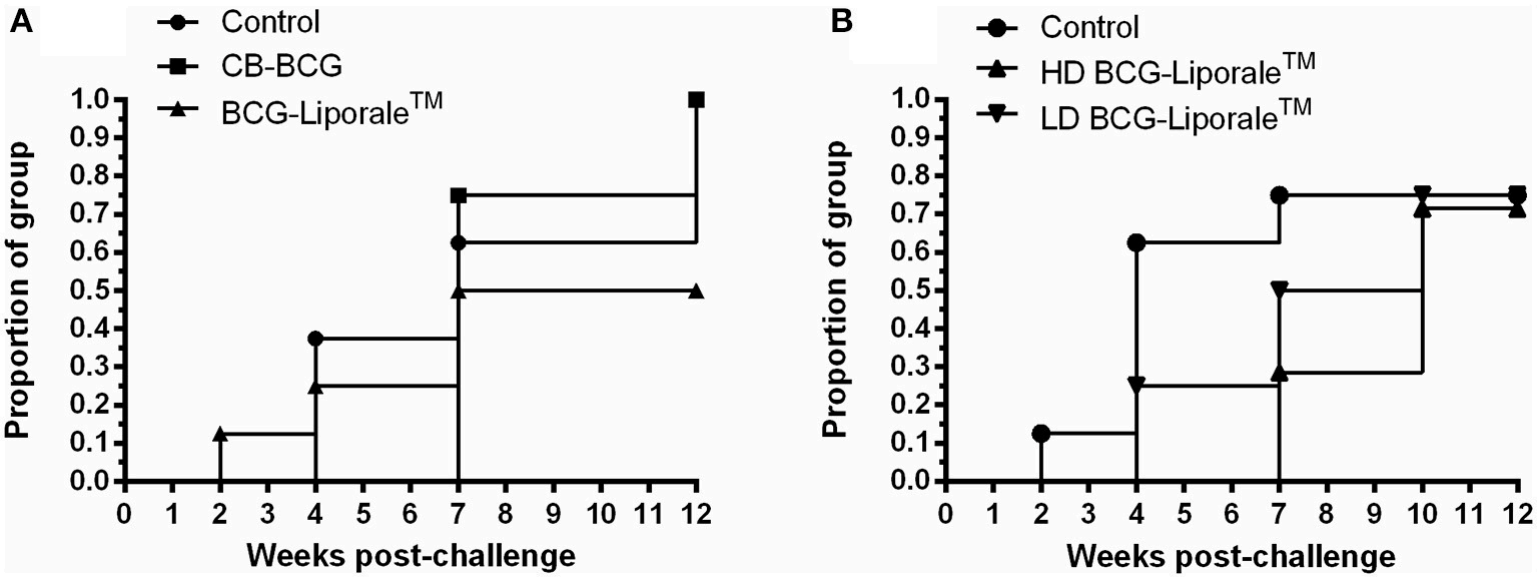
**Vaccination of badgers with BCG and challenged 13 weeks later with endobronchial ***M. bovis***: Proportion of each treatment group positive in the Brock TB Stat-Pak serological test after challenge with ***M. bovis*** from experiments VES3 (A)** and VES4 **(B)**. Badgers were vaccinated with either CB-BCG (■), HD BCG-Liporale™ (▲) or LD BCG-Liporale™ vaccine (▼). Liporale™ alone was used as a negative control for vaccination (•).

In VES3, vaccination with HD BCG-Liporale™ resulted in significantly fewer Stat-Pak positive badgers after challenge compared with the other groups (*p* = 0.0235, Chi-square test) and vaccination caused a significant delay to the seroconversion of those that did become positive (*p* = 0.008, Log-rank test) (Figure 5A). In VES4, vaccination with BCG-Liporale™ at either dose did not result in significantly fewer Stat-Pak positive badgers after challenge compared with the controls. Although the median time taken to seroconversion after challenge was 4 weeks in the case of controls and delayed to 8.5 and 10 weeks for high and low dose vaccination groups, respectively, these differences were not significant (Log-rank test) (Figure 5B). One VES4 control animal was reactive in the Brock TB Stat-Pak for the duration of the study (data not shown). This animal had low PPD-B responses pre-challenge but no response to ESAT-6/CFP-10 pre-challenge, and the severity of TB *post-mortem* was typical of the group, suggesting it was genuinely uninfected at the start of the study. No other badger was seropositive before vaccination. One HD BCG-Liporale™ vaccinated animal (in VES3) became reactive in the Stat-Pak 7 weeks after vaccination and remained so for the duration of the experiment (data not shown).

## Discussion

In both studies we demonstrated considerable levels of protection against the development of tuberculosis in badgers vaccinated with BCG. However, there was a surprising result: the high dose of BCG was protective in the first but not the second study, whereas the low dose of BCG was protective in the latter.

We assessed the severity of disease in our experimental challenge model using a combination of visible pathology (number and severity of visible lesions expressed as a total Visible Lesion Score), histopathology (granuloma severity, collagen abundance), and dissemination of *M. bovis* (by culture and histopathology) and the excretion of *M. bovis*. In VES3, the oral administration of 1.86 × 10^8^ CFU BCG in Liporale™ gave statistically significant protection in every measure used to assess vaccine efficacy, with the exception that it did not significantly reduce the frequency of *M. bovis* excretion by badgers or the bacterial load per gram of tissue submitted for bacteriology. For all the measures of gross disease, the CB-BCG vaccine resulted in less severity than the controls. For both of the histological measures (granuloma severity and collagen deposition) these reductions were statistically significant. Due to the small number of badgers used in the CB-BCG group, statistical significance was not demonstrated in the number of tissues infected.

In VES4 there was the unexpected result that the lower dose was more protective than the higher dose. Although in the HD BCG-Liporale™ group there were three parameters with better results than the controls, the differences were not significant statistically. One explanation for this is the small number of badgers used (*n* = 4), exacerbated by the variability of the response within each of the groups. Indeed there appeared to be greater variation in the disease parameters seen in the control group of VES4 compared with the control group of VES3. Whilst this is nothing beyond which we have seen previously in this model, it does illustrate the difficult in conducting experimental efficacy studies using outbred animals where there is a practical constraint on group sizes, as is the case here. Nonetheless, it is still clear, not only from the disease severity score but from additional measures, that BCG vaccination altered the expression and progression of experimental infection. There was evidence in both studies that BCG in Liporale™ was able to restrict the ability of *M. bovis* to disseminate from the thoracic cavity, the initial site of infection, even if it didn't apparently reduce the bacterial load within the tissue samples. These results are in general agreement with the protection seen in two earlier assessments of oral BCG in the same lipid matrix (Corner et al., 2010; Murphy et al., 2014) where the impact on bacterial load was minimal. Excretion of *M. bovis* was intermittent in both studies, and the impact of vaccination on this measure is less clear than in earlier studies when injectable vaccination was performed (Lesellier et al., 2011).

Failure of oral BCG to reduce the apparent bacterial load or excretion in the badger challenge model could be an artifact of the model in which the *M. bovis* challenge dose is likely to be higher than encountered in natural infection. Both for experimental and natural infection, excretion of *M. bovis* is highly intermittent (Chambers et al., 2002; Lesellier et al., 2011). Furthermore, the quantification of *M. bovis* in tissues is only semi-quantitative at best since counts were either derived either from the majority of the organ (e.g., in the case of lymph nodes) or a far smaller proportion of the organ (e.g., spleen). It remains the case, however, that at the doses of BCG used in these studies and/or the method of delivery that the efficacy of the vaccine does not extend to reducing bacterial load. Whether this will ultimately reduce the efficacy of oral vaccination in the field will need to be determined once the minimum efficacious dose of oral BCG has been established experimentally. Whilst a reduction in bacterial load through vaccination would be ideal it might not be essential to prevent onward transmission of infection; which is a combination of bacterial load and the opportunity afforded for excretion by tissue location and pathology. We found previously, through thorough histopathological examination of naturally-infected badgers, that both mildly and severely infected badgers have the potential to excrete *M. bovis* by several routes (Gavier-Widen et al., 2001).

All animals responded in the IFNγ ELISPOT after they were challenged with *M. bovis*. Responses to ESAT-6/CFP-10 antigens were seen after challenge but the level of IFNγ production correlated poorly with disease severity. The same poor association has been found in cattle (Vordermeier et al., 2002), and in a previous badger study with oral BCG (Murphy et al., 2014). In VES3, responses to PPD-B in the ELISPOT assay were seen in control animals prior to challenge. As the response of all animals from all treatment groups in VES3 had higher responses to PPD-A by ELISPOT in the run up to challenge (data not shown), we conclude from this that the PPD-B responses were most likely a cross-reaction to exposure with *Mycobacterium avium* from the environment. As birds have access the pens in which the badgers are housed prior to challenge, there is opportunity for this to occur. As prior to challenge no responses were seen in these animals to ESAT-6/CFP-10 and *M. bovis* was not isolated from clinical samples, we are confident that none of the VES3 animals were harboring TB prior to challenge.

We also examined the impact of oral BCG on the serological response of badgers to infection. Vaccination with HD BCG-Liporale™ in VES3 reduced the extent of seroconversion after challenge, consistent with previous observations for BCG given subcutaneously (Lesellier et al., 2009), but not for BCG given intramuscularly (Lesellier, unpublished results). In VES4, a reduction in the extent of seroconversion was not observed following vaccination with BCG-Liporale™ at either dose. One VES4 control animal was reactive in the Brock TB Stat-Pak for the duration of the study. Importantly, this animal was consistently negative by IFNγ and culture before enrolment in the study and no *M. bovis*, other than the challenge strain, was isolated from this animal. We therefore conclude the sero-reactivity of this animal was non-specific, possibly due to infection with environmental mycobacteria with cross-reacting antigens.

BCG was not cultured from the feces collected from the animal pens after vaccination in either experiment. This differed from a previous study in which BCG was recovered infrequently (on three occasions; three and 17 days after vaccination) from the feces of badgers vaccinated with 10^8^ CFU (Corner et al., 2010) but in low concentrations (≤ 20 CFU/g feces). That is, following oral vaccination with BCG either un-encapsulated or encapsulated in lipid, excretion from the alimentary tract occurs at low frequency and low quantity. This low or absence of excretion indicates either very efficient uptake of BCG by mucosal-associated lymphoid tissue (MALT) or a substantial loss of BCG viability as it transits the gastrointestinal tract. To understand these processes better, we are conducting studies to evaluate BCG survival in an artificial gut that simulates the conditions of the badger stomach and small intestine, along with *in vivo* studies to evaluate the uptake of orally administered BCG from the proximal and distal parts of the alimentary tract of badgers. Although BCG encapsulated in lipid has previously been shown to extend the survival of BCG *in vivo* (Aldwell et al., 2006), the persistence of BCG in organs/tissues or BCG excretion was apparently not associated with enhanced protection in our studies.

We sought an explanation for the difference in the efficacy of HD BCG-Liporale™ in VES3 and the low dose in VES4 which were protective and the unexpectedly poorer performance of the high dose in VES4. It is possible that differences between the protection induced by HD BCG-Liporale™ in VES3 and VES4 is simply a consequence of the small number of animals in the VES4 group (*n* = 4). Whilst it is clear oral vaccination with BCG can be highly efficacious, it may be inherently more variable in its efficacy than when administered parentally. The high dose of vaccine in both studies was biologically equivalent and the experimental infections very similar based on disease in the control groups, so these results may have been a matter of chance resulting from the small group size.

It has been assumed that the most significant contribution of the lipid matrix is to protect BCG from inactivation in the stomach and to enhance the uptake of viable BCG from the small intestine (Aldwell et al., 2006). Consistent with this, the ISL lipid formulation has been demonstrated to confer protection superior to unformulated oral BCG in guinea pigs (Clark et al., 2008; Vipond et al., 2008) and possums (Aldwell et al., 2003). However, the results presented here, and data we have generated from guinea pig studies in which BCG administered orally in Liporale™ was recovered from cervical LNs (Clark et al., 2008), suggest that oropharyngeal uptake of orally-presented BCG may be important for stimulating protective immunity. Evidence for a protective effect of the un-encapsulated CB-MSG in VES3 suggests this may be the case. Although CB-BCG only significantly reduced histological measures of disease severity, the reduction in pathology was still encouraging given that we only included four badgers in this group.

The objectives of the two (VES) reported here were to compare the efficacy of BCG Danish strain in a lipid matrix with unformulated BCG given orally and gain insights into the impact that vaccine dose has on efficacy. We achieved those objectives. Ultimately BCG will need to be delivered to wild badgers in bait and we are making progress toward that objective (Robertson et al., 2016). For the purposes of this study it was important that we could control the dose of BCG each badger received so the animals were dosed manually. In the wild it will not be possible to control the amount of BCG each animal consumes and field studies to evaluate this will be required before a product can be licensed for use. At this point in time, further research is still needed before we are able to recommend a final dose of BCG for oral vaccination of badgers against TB or to know whether oral vaccination of wild badgers with BCG will significantly reduce transmission of the disease.

## Conclusions

The data presented here, together with previous reports (Corner et al., 2010; Murphy et al., 2014), demonstrate that the oral administration of BCG in ISL Liporale™ matrix significantly reduced the extent of tuberculous lesions following intrabronchial *M. bovis* challenge. The challenge dose used is likely to be higher than that which occurs during natural exposure to infection. Our experimental challenge is a more stringent test of vaccine efficacy than might be encountered naturally.

## Author contributions

MC and SL designed and drafted the work, and with assistance from LC, analyzed and interpreted the data. GW, SP, SG, RA, DJD, DD, UW, FS, AN, AKN, TC, LC, and SL conducted the work. GW, SP, SG, RA, DJD, DD, UW, FS, AN, AKN, TC, and LC assisted MC with drafting the work. All authors were involved in critical review of the work, gave their final approval of the version to be published, and agree to be accountable for all aspects of the work in ensuring that questions related to the accuracy or integrity of any part of the work are appropriately investigated and resolved. MC is Professor of Veterinary Bacteriology, School of Veterinary Medicine, Faculty of Health & Medical Sciences, University of Surrey, United Kingdom where he is funded 0.4 through the Higher Education Funding Council for England (HEFCE).

### Conflict of interest statement

The authors declare that the research was conducted in the absence of any commercial or financial relationships that could be construed as a potential conflict of interest. FA is Chief Scientist and Director of Immune Solutions Ltd, the company that supplied the vaccine. Relevant patents derived by Immune Solutions from PCT/NZ2002/000132, entitled “Antigenic compositions” are as follows. Granted in Australia (2008202282), Canada (2,454,920), China (837462), India (235505), Japan (4685350), New Zealand (562036), South Africa (2004/1211), and United States (7,758,869). Pending in Europe (02760915, 10188891) and Hong Kong (04109263.7).
